# The Effect of *Rhizophagus intraradices* on Cadmium Uptake and *OsNRAMP5* Gene Expression in Rice

**DOI:** 10.3390/ijms26041464

**Published:** 2025-02-10

**Authors:** Xiaoqi Bao, Junli Liu, Gaoyang Qiu, Xiaodong Chen, Junbo Zhang, Hua Wang, Quan Zhang, Bin Guo

**Affiliations:** 1 College of Environment, Zhejiang University of Technology, Hangzhou 310021, China; 13868984157@163.com; 2 State Key Laboratory for Quality and Safety of Agro-Products, Institute of Environment, Resource, Soil and Fertilizers, Zhejiang Academy of Agricultural Sciences, Hangzhou 310021, China; liujunli@zaas.ac.cn (J.L.); qiugy@zaas.ac.cn (G.Q.); chenxiaodong@zaas.ac.cn (X.C.); zhangjunbo@zaas.ac.cn (J.Z.); 3 State Key Laboratory for Quality and Safety of Agro-Products, Institute of Virology and Biotechnology, Zhejiang Academy of Agricultural Sciences, Hangzhou 310021, China; wanghua3@hotmail.com

**Keywords:** arbuscular mycorrhizal fungi, cadmium, uptake, *OsNRAMP5*, rice

## Abstract

The molecular mechanism of arbuscular mycorrhizal fungi (AMF) in reducing cadmium (Cd) accumulation in plants remains unclear. In this respect, the effects of *Rhizophagus intraradices* (Ri) inoculation under Cd stress on rice growth, the uptake of Cd along with other elements, and the expression of Cd transport genes, including *OsNRAMP1/5*, were studied using wild-type (WT) and *osnramp5* mutant rice. The results showed that Ri inoculation did not affect rice growth. The uptake of Cd of the *osnramp5* mutant was much lower than the WT, as 27.6%, 17.5%, and 39.9% of Cd were noted in the grains, shoots, and roots, respectively. For the WT, Cd alone significantly promoted the *OsNRAMP5* expression in shoots, but Ri inoculation significantly suppressed *OsNRAMP5* expression and significantly reduced its grain and shoot Cd by 44.4% and 62.3%, respectively, compared to the Cd alone treatment. In contrast, for the *osnramp5* mutant, Ri inoculation did not influence *OsNRAMP5* expression or the grain and shoot Cd. Furthermore, the expression of other Cd transporters (*OsIRT1*, *OsZIP3/7*, *OsCAX1a*) in both varieties were not changed under the treatments. In conclusion, Ri inoculating significantly reduced Cd uptake by rice, with the molecular mechanism by negative regulation of expression of the *OsNRAMP5* gene.

## 1. Introduction

The food safety issues caused by excessive cadmium (Cd) in rice have posed a serious threat to public health and safety [1,2,3]. One of the effective and important strategies to mitigate rice Cd risk is regulating the expression of Cd-related transport proteins in rice (*Oryza sativa* L.), thereby reducing Cd accumulation in the grains. *OsNRAMP5* (natural resistance-associated macrophage protein, NRAMP) is an important Cd transport gene in rice. Knockout of *OsNRAMP5* studies have shown that the loss of this gene significantly reduces Cd accumulation in the roots and grains [4], while the overexpression of *OsNRAMP5* promotes Cd transport into root cells but significantly decreases Cd accumulation in the aerial parts and grains of rice [5]. In addition to *OsNRAMP5*, its family member *OsNRAMP1* also participates in the absorption of multiple elements, including Cd, Mn, Fe, and As, in rice [5]. Additionally, Cd transport genes in rice include *OsIRT1* (Iron-regulated transporter, IRT), which is mainly expressed in the phloem, stems, and leaves of rice [6] and participates in the transport of Zn and Cd within rice [7,8]; rice Zn-regulated transporter, *OsZIP3* (Zn-regulated transporter, ZIP), which is highly expressed in the nodes of rice [9] and is responsible for the transport of Cd from the roots to the aerial parts of the plant [10]; *OsZIP7*, which plays a role in loading in the xylem of rice roots and in the transport between the vascular bundles of the internodes, delivering Zn/Cd to the grains [11]; and *OsCAX1a* (Cation^2+^/H^+^ exchanger, CAX), which is primarily expressed on the vacuolar membrane and is responsible for Cd absorption [12].

Arbuscular mycorrhizal fungi (AMF) can form symbiotic relationships with the roots of most plants [13,14], playing an important role in regulating plant heavy metal uptake [15,16,17]. On the one hand, AMF can effectively expand the range of plant root absorption [18] by activating mineral nutrients and heavy metals in soils [19,20]. On the other hand, AMF can alleviate the toxicity of heavy metals within plants via several mechanisms, such as strengthening antioxidant defense [21], regulating related transport genes [22], controlling the metal compartmentalization or dilution of metals within plant tissues [23], and facilitating the formation of metal complexes. Studies have shown that AMF suppresses Cd accumulation in rice leaves by downregulating the expression of genes such as *OsLCT1*, *OsHMA9*, *OsHMA2*, and *OsZIP3* [24]; the key genes for Cd transport at the rice stem nodes, such as *OsIRT1* and *OsHMA2*, may also be regulated by AMF [19]. The effect of AMF on rice Cd absorption may also be related to the regulation of *NRAMP* gene expression. For example, AMF (*Glomus versiforme*, Gv) can significantly downregulate the expression of *OsNRAMP5*, thereby reducing Cd transport in both the roots and aerial parts of drought-resistant rice [25]. Inoculation with two types of AMF (Ri, and *Funneliformis mosseae*, Fm) significantly reduces Cd concentrations in both the roots and aerial parts of rice; however, Ri treatment significantly inhibits *OsNRAMP5* expression in the roots, while Fm treatment significantly promotes it [26]. Therefore, the main transport genes that govern AMF’s effect on rice Cd absorption have not been fully elucidated.

*Rhizophagus intraradices* is a typical arbuscular mycorrhizal fungus that has been widely used to promote plant growth and stress resistance [21,27,28,29]. The use of CRISPR/Cas9-generated mutants is an effective biological research method that allows for the precise localization and validation of specific key functional genes, enabling scientific inference on the roles of mutated genes within organisms. In this study, we selected two rice materials, the knockout of *OsNRAMP5* (*osnramp5* mutant, *P*) and wild-type rice (WT), to investigate the effects of AMF inoculation on rice Cd absorption and the regulation of related transporters. The aim is to elucidate the molecular mechanisms by which AMF influences Cd absorption in rice and provide a theoretical basis for the technological application of AMF in reducing Cd accumulation in rice.

## 2. Results

### 2.1. Mycorrhizal Colonization

**Figure 1:**
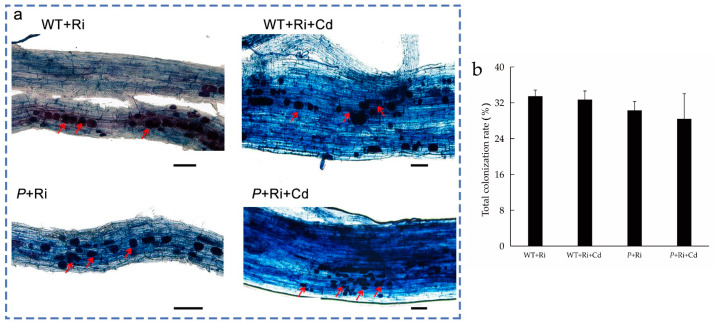
The colonization picture (**a**) and total colonization rate (**b**) of the WT and *P* plant roots under different treatments (bar = 50 μm). “WT” indicates wild-type rice grown under normal conditions; “*P*” indicates the *osnramp5* mutant grown under normal conditions; “WT+Ri” indicates wild-type rice inoculated with Ri; “WT+Cd” indicates wild-type rice subjected to Cd stress; and “WT+Ri+Cd” indicates wild-type rice inoculated with Ri under Cd exposure. The “*P*+Ri” *osnramp5* mutant was inoculated with Ri; the “*P+*Cd” *osnramp5* mutant was subjected to Cd stress; and the “*P*+Ri+Cd” *osnramp5* mutant was inoculated with Ri under Cd exposure. The red arrow indicates the arbuscules.

As shown in Figure 1 and Figure S1, no mycorrhizal colonization was observed in the rice roots when Ri was not added to the soil. After the addition of exogenous Ri to the soil, mycorrhizal colonization was observed at the root tips of all treatments, with a colonization rate of approximately 30%. No significant differences in the colonization rate were observed between the treatments with or without Cd or among the different rice varieties.

### 2.2. Biomass

**Figure 2:**
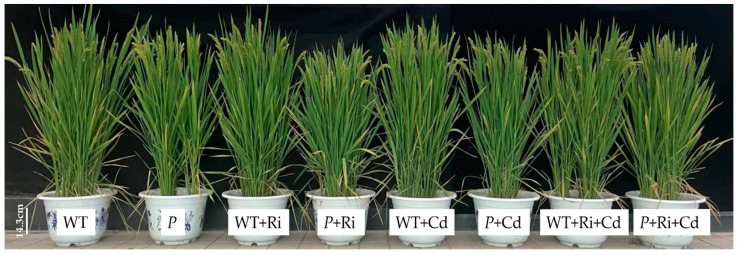
Comparison of the growth status of WT and *P* at maturity under different treatments (bar = 14.3 cm). The abbreviations used in Figure 2 were same as that of Figure 1.

Under the exogenous Ri treatment alone, there were no significant differences in plant height, grain weight, or the dry weight of aerial parts and roots between the WT and *P mutant* compared to the control (Figure 2 and Table 1). However, the root length of the *P* treated with Ri was significantly greater than that of the control. Under the exogenous Cd treatment alone, the grain dry weight of both rice varieties significantly increased, and the dry weight of the aerial parts of the WT also significantly increased. Under the combined Ri and Cd treatment, except for a significant increase in the grain dry weight of the WT, no significant differences were observed in plant height, grain weight, the dry weight of aerial parts and roots, or root length compared to the untreated controls.

### 2.3. Cadmium Uptake by Rice

**Figure 3:**
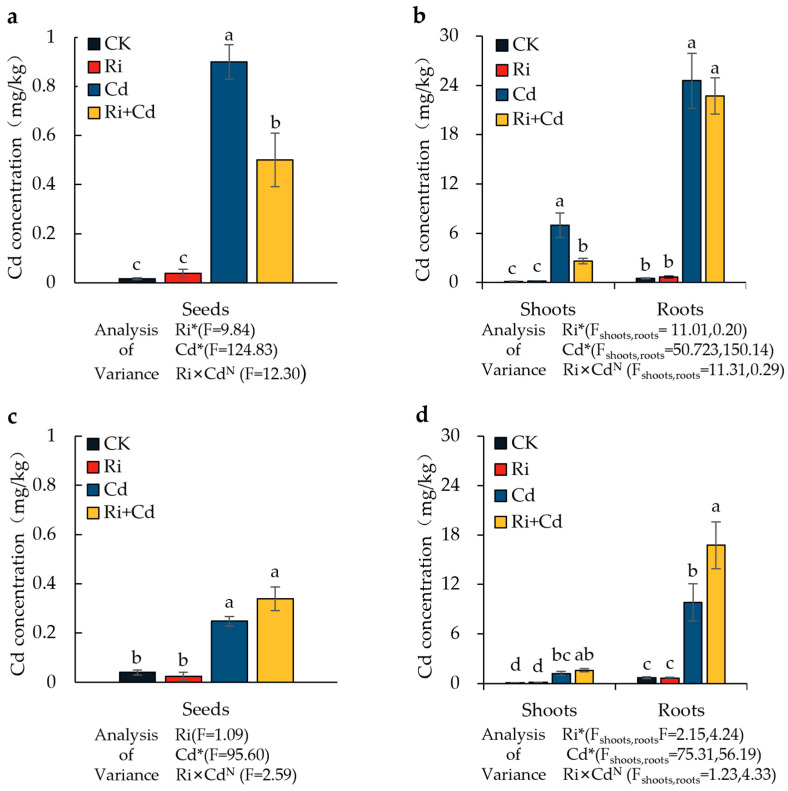
The Cd concentration in seeds, shoots, and roots of the WT (**a**,**b**) and *P* (**c**,**d**) plants under the Ri and Cd treatments. For the data (means ± SEs, *n* = 5), different letters (a, b, c, d) indicate significant differences (*p* < 0.05, Duncan’s test). A two-way ANOVA was used to test the interaction between the Ri treatment and Cd pollution. N: not significant, * *p* < 0.05. The abbreviations used in Figure 3 were same as that of Table 1.

Under the Cd treatment, the Cd concentrations in the grains, aerial parts, and roots of both rice varieties significantly increased compared to the control. Cd primarily accumulated in the rice roots, followed by the aerial parts, with the grain Cd concentration being only about 2% of that in the roots (Figure 3). For the wild-type rice (WT), the grain Cd in the Ri-inoculated rice was significantly lower than that in the non-inoculated treatment, with a reduction of 44.4%. The Cd concentration in the aerial parts also significantly decreased, with a reduction of 62.3%, while the root Cd concentration showed no difference. The Cd level in all parts of the mutant (*P*) was much lower than in the wild-type rice, with Cd concentrations in the grains, aerial parts, and roots being only 27.6%, 17.5%, and 39.9%, respectively, of those in the wild-type rice. Unlike the WT, the Cd concentration in the roots of the *P* significantly increased after Ri inoculation, with a 71% increase compared to the non-inoculated treatment. There were no significant differences in the Cd concentration of the aerial parts and grains between the inoculated and non-inoculated treatments.

### 2.4. Uptake of Other Essential Elements in Rice

Cd significantly affected the uptake of essential elements such as K, Fe, Ca, and Zn in the rice (Table 2). For the WT, Cd stress alone significantly increased the K concentration in the grains and roots, as well as the Ca level in the shoots. The Ri treatment alone significantly increased the Ca and Fe concentration in the shoots. The Ri combined with Cd treatment significantly increased the grain K and Ca, the shoot Zn, and the root K and Zn. In contrast, for the *P*, Cd stress alone significantly increased the grain K and the root Ca and Fe. The Ri treatment alone significantly increased the grain K, the shoot Ca, and the root Zn but decreased the root K. Under the combined Ri and Cd treatment, the K concentration in all parts of the rice increased.

### 2.5. Cadmium Transport Gene Expression in Rice Leaves

**Figure 4:**
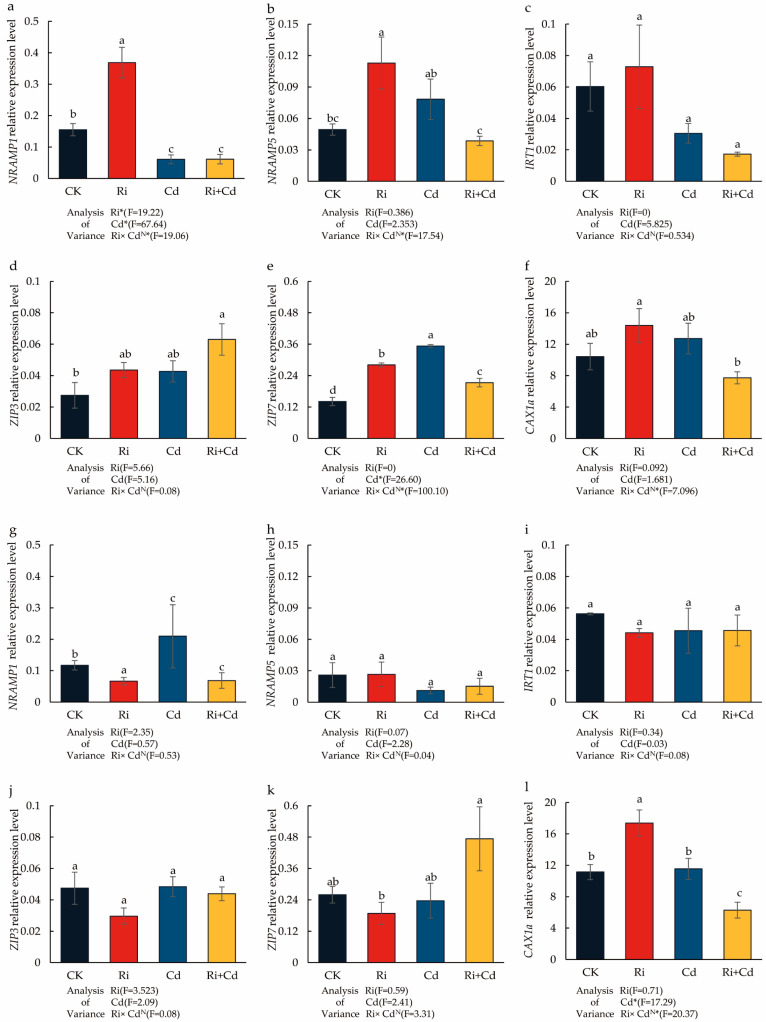
The impact of the Ri and Cd treatments on the relative expression level of Cd transporters in the WT (**a**–**f**) and *P* (**g**–**l**) plants. Leaves were harvested separately for qRT-PCR analysis. Fold changes in the gene expression were normalized against the reference gene *OsACTIN*. For the data (means ± SEs, *n* = 5), different letters (a, b, c, d) indicate significant differences (*p* < 0.05, Duncan’s test). A two-way ANOVA was used to test the interaction between the Ri treatment and Cd pollution. N: not significant, * *p* < 0.05. The abbreviations used in Figure 4 were same as that of Table 1.

In the Cd-free condition, the *OsNRAMP1* and *OsNRAMP5* expression of the WT was significantly upregulated after Ri inoculation, with the expression levels increasing by 2.38 and 3.16 times, respectively, compared to the control (Figure 4). The Cd treatment alone also significantly promoted the expression of the *OsNRAMP5* gene in the rice leaves, increasing its expression by 2.19 times. However, it significantly reduced the expression of *OsNRAMP1*, which was only 0.39 times the expression level of the control. In contrast to the results with Ri inoculation alone, the co-treatment with Ri and Cd significantly downregulated the expression of both *OsNRAMP1* and *OsNRAMP5*, with their expression levels being only 40% and 59% of the control treatment, respectively. For the other genes, there were no significant differences in the expression of the *OsIRT1* gene across treatments. Under Cd stress, the expression of *OsZIP7* was significantly upregulated, but its expression was inhibited after mycorrhizal inoculation. Under the co-treatment with Ri and Cd, the expression of the *OsZIP3* gene was significantly higher than the control treatment, while the expression of *OsCAX1a* was significantly lower than in the Cd treatment alone.

For the *P*, there were no significant differences in the expression of the *OsNRAMP1/5* genes across treatments, nor in the expression of *OsIRT1* and *OsZIP3*. Under the co-treatment with Ri and Cd, the expression of the *OsZIP7* gene was significantly higher than in the Cd treatment alone. Ri inoculation alone significantly promoted the expression of the *OsCAX1a* gene, but under the co-treatment with Ri and Cd, its expression was significantly lower than the control.

## 3. Discussion

Under Cd stress, the Ri colonization rate of the WT and *osnramp5* mutant remained around 30%, with no significant difference compared to the control (no Cd treatment). This result is consistent with the findings of [30], indicating that the Ri used in this study has strong tolerance to Cd toxicity [31,32]. Mycorrhizal inoculation had no significant effect on the growth of wild-type rice or the mutant, nor on the uptake of macronutrients such as K. This may be because the level of soil nutrients used in this experiment was relatively high (available phosphorus 25 mg/kg, alkali-hydrolyzable nitrogen 180.6 mg/kg). Generally, it is difficult to detect the growth-promoting effect induced by AMF when plants are grown in high soil fertility environments [15,33,34].

After Ri inoculation, the movement of Cd from the rice roots to the shoots was significantly inhibited in the WT. The Cd concentration in the shoots and grains of the Ri treatment was only 37.7% and 55.6%, respectively, of that in the non-inoculated Cd treatment, indicating that AMF significantly regulates the transport of Cd within the rice plant. Similar results have been observed in rice with the inoculation of different AMF species, such as Ri [19,26,35], Fm [24,26,35,36], and Ri [30,37]. For example, under 2 mg/kg and 10 mg/kg Cd stress, the Cd concentration in rice seeds inoculated withRi was only 79% and 68% of that in the non-inoculated treatment [30]. Some research suggested that the lower Cd accumulation by AMF inoculation might be due to the reconstruction of the root cell wall and, thereby, increased Cd fixation [35,36,37]. Comparatively, other research speculated that AMF inoculation affected the transporters of Cd in rice [19,26,38]. This study proved that AMF can exert a significant regulatory effect on *OsNRAMP5* in rice shoots. In the absence of Cd, its expression was upregulated, whereas under Cd stress, it was downregulated. OsNRAMP5 is a membrane-localized transporter with transmembrane domains, playing a crucial role in the absorption and transport of Fe, Mn, and Cd in rice [39,40,41,42]. The knockout of *OsNRAMP5* exhibited defects in Cd absorption by the roots and its transfer to the aerial parts, resulting in only 8–27% of the Cd concentration in the aerial parts and roots compared to the conventional materials [4,43].

To further validate the results, the *osnramp5* mutant was also tested in this study. The results showed that the Cd concentrations in the seeds, aerial parts, and roots of the mutant were only 27.6%, 17.5%, and 39.9% of those in the conventional rice, respectively. Moreover, the expression of *OsNRAMP5* in the aerial parts was significantly lower than in the WT, and there were no significant differences in *OsNRAMP5* expression across treatments, indicating that *OsNRAMP5* is a key gene controlling Cd transport in rice. Since Ri inoculation had no significant effect on *OsNRAMP5* gene expression in the mutant, there was no significant difference in Cd concentration in the shoots compared to the non-inoculated treatment. These results demonstrate that under Cd stress, mycorrhizae negatively regulate the expression of *OsNRAMP5* in the aerial parts of rice, thereby significantly inhibiting the transport of Cd from the roots to the aerial parts. The results are consistent with those reported by Zhu et al. in upland rice [25]. Additionally, under mycorrhizal inoculation, the Cd concentration in the mutant roots was significantly higher than in the non-inoculated mutants. This may be related to the increased root absorption area by the extension of the fungi or changes in the structure of the rice root cell wall [37,44,45,46]. These findings provide a new insight into the role of AMF on Cd uptake by rice plants and highlight the need for further research on the contribution of AMF in field studies in the future.

## 4. Materials and Methods

### 4.1. Experimental Materials

The AMF inoculant used in this study was Ri (BJ09), provided by the Beijing Academy of Agricultural and Forestry Sciences. The rice varieties used were the wild-type rice (*Oryza sativa* L.cy Huajing1) and the *osnramp5* mutant (*P*), provided by the Zhejiang Academy of Agricultural Sciences. The mutant material was generated using the CRISPR/Cas9 knockout system to design the target site [4]. The experimental soil was collected from the plow layer of paddy fields in Jiaxing, Zhejiang, with the following properties: pH 5.21, organic matter 28.77 g/kg, total nitrogen 1.7 g/kg, available phosphorus 25.0 mg/kg, available potassium 92.25 mg/kg, alkali-hydrolyzable nitrogen 180.6 mg/kg, and total Cd 0.15 mg/kg. After air-drying, the soil was passed through a 2 mm sieve and sterilized at 120 °C for 60 min before use.

### 4.2. Experimental Design

Rice seeds were disinfected with 30% (*v*/*v*) H_2_O_2_ for 10 min and then rinsed repeatedly with deionized water and soaked for 72 h until germination, with water changes three times during the process. Seeds with consistent germination were selected, placed on rice culture floating boards with small holes, and further cultivated in 1/3 strength Kimura B nutrient solution for seedling growth. The nutrient solution was replaced every 7 days. When the rice seedlings reached the two-leaf and one-heart stage, uniform seedlings were selected and transplanted into the soil.

The rice pot experiment was conducted in a greenhouse, with the temperature controlled between 25 °C and 35 °C. Pots (Φ38 × 26 cm) were filled with 10 kg of soil. Prior to planting, urea, KH_2_PO_4_, and K_2_SO_4_ were applied as base fertilizers in the amounts of N: P_2_O_5_: K_2_O = 0.2: 0.15: 0.2 (g/kg). The experiment included 8 treatments: wild-type rice (WT), WT with Ri inoculation (WT+Ri), WT with Cd treatment (WT+Cd), WT with Ri inoculation and Cd treatment (WT+Ri+Cd), mutant material (*P*), *P* with Ri inoculation (*P*+Ri), *P* with Cd treatment (*P+*Cd), and *P* with Ri inoculation and Cd treatment (*P*+Ri+Cd). Each treatment had 5 replicates, totaling 40 pots, arranged randomly. Ri inoculation was performed by thoroughly mixing 200 g of Ri inoculant with 10 kg of soil before rice transplantation. For the exogenous Cd treatment, a small amount of reserve soil (1.0 kg) was mixed with 1 L of a high-concentration Cd solution containing 5000 mg/L, air-dried, sieved, and aged for 3 months before being mixed with uncontaminated reserve soil to adjust the Cd concentration to 2 mg/kg. The rice was managed under continuous flooding and harvested after 120 days of cultivation until maturity. After thorough washing with deionized water, the rice plant height, root length, and yield were measured. The rice samples were divided into three parts: grains, aerial parts, and roots. They were dried at 70 °C to constant weight, weighed, and then ground through a 2 mm sieve for the analysis of Cd and other elements. Fresh rice leaf samples were also collected for RNA extraction and gene expression analysis.

### 4.3. Sample Determination

**Colonization rate determination**: The analysis of mycorrhizal colonization was carried out using the magnified intersection method described by Xue et al. [47,48] with slight modification. Fresh root samples were cut into 1 cm segments. More than 100 root fragments were decolorized in a 10% NaOH solution at 90 °C for 1 h until transparent, neutralized in a 2% HCl solution for 6 min, and then stained with trypan blue. Then, 25 root segments were placed on each microscopic slide, and four slides were made for each seedling. Using 10× object magnification (OLYMPUS DP74, Tokyo, Japan), 50 views per slides and 200 views per biological replicate were random surveyed and classified into five groups: no colonization, hyphae with arbuscules (A), hyphae with arbuscules and vesicles (B), only hyphae (C), and hyphae with vesicles (D). Five biological replicates were counted for each treatment. The percentages of each group were calculated by the number of each sector divided by total views. The total colonization rate was calculated using the following formula:

Total colonization rate= A% + B% + C% + D%.

**Element analysis**: First, 0.500 g of rice grains and 0.250 g of rice stems, leaves, and roots were placed in digestion tubes and then soaked overnight with 5 mL of HNO_3_. The samples were digested using a microwave digestion system (CEM, Charlotte, NC, USA) at 120 °C for 10 min, followed by a temperature increase to 160 °C for 10 min, and finally maintained at 190 °C for 30 min until the solution became clear [35]. The volume was then adjusted to 50 mL. The Cd content was measured using an inductively coupled plasma mass spectrometer (Thermo Fisher Scientific, Waltham, MA, USA), while K, Ca, Fe, Zn, and other medium and macronutrient elements were determined using an inductively coupled plasma optical emission spectrometer (Teledyne Leeman Lab, Mason, OH, USA).

**RNA extraction and gene expression analysis**: Total RNA was extracted from the root samples using a plant total RNA extraction kit (Vazyme, Nanjing, China). RNA was reverse transcribed into cDNA using HiScript II Q RT SuperMix for qPCR (Vazyme, Nanjing, China). Gene expression levels were quantified using ChamQ Universal SYBR qPCR Master Mix (Vazyme, Nanjing, China). The 15 μL quantitative reaction system included 1.5 μL cDNA, 0.3 μL primers (10 μM), 7.5 μL 2 × ChamQ Universal SYBR qPCR Master Mix, and 5.6 μL ddH_2_O. The reaction was initiated with denaturation at 95 °C for 30 s, followed by 40 cycles of 95 °C for 10 s, 60 °C for 30 s, and 72 °C for 30 s. After the cycles, a melt curve analysis (65–95 °C) was performed to check amplification specificity. The samples were analyzed using a LightCycler480 (Roche, Switzerland). In our previous research, we compared the transcriptional expression levels of *OsZIP3* genes in wild-type rice using three reference genes (*OsACTIN*, *OsGAPDH*, and *OsUbiquitin*) and two formulas (2^(−∆Ct)^ and 2^−∆∆Ct^) [49,50,51,52,53,54] inoculated with or without AMF under Cd stress. As shown in Figures S2 and S3, the expression level of the same gene in the same treatment was different when different reference genes and formulas were used, but compared to the control, the expression trend of the *OsZIP3* genes was the same in the rice inoculated with AMF under Cd stress when using different reference genes and formulas. Furthermore, the analysis of melting curves and the amplified curve for these genes (*OsACTIN*, *OsGAPDH*, *OsUbiquitin*, and *OsZIP3*) showed that the expression level of these genes was accurate and reproducible (Figures S4 and S5). Therefore, it was assumed that the reference gene (*OsACTIN*) was stably expressed, and the formula used was suitable for calculating the relative expression levels in this study. The relative gene expression levels were calculated using the 2^(−∆CT)^ method [12,54] as follows:

Target gene relative expression levels = Power (2 − (Ct_target gene_ − Ct_internal reference gene_ Mean)), Ct: computerized tomography. Different target gene expressions were normalized against the same internal reference gene (*OsACTIN*).

All primer sequences used are shown in Table 3 and Table S1.

### 4.4. Statistical Analysis

Statistical analysis was performed using IBM SPSS Statistics 27. All data were expressed as mean ± standard error (mean ± SE); different letters indicate significant differences (*p* < 0.05, Duncan’s test). A two-way ANOVA was used to test the interaction between the Ri treatment and Cd pollution. Graphs were created using Excel 2016 and Origin 2021.

## 5. Conclusions

*Rhizophagus intraradices* inoculation had no significant effect on the growth of the WT and the *osnramp5* mutant rice. Under Cd stress, Ri inoculation significantly reduced the expression of *OsNRAMP5* in the shoots of the WT, with the expression level being only 59% of that in the control treatment. As a result, the Cd concentration in the seeds and aerial parts also significantly decreased, with reductions of 44.4% and 62.3%, respectively. Comparatively, Ri inoculation failed to affect the *OsNRAMP5* expression and Cd concentration of the *osnramp5* mutant shoots. In brief, Ri inoculation can significantly reduce Cd accumulation in rice, with the molecular mechanism involving the negative regulation of the *OsNRAMP5* gene expression in the aerial parts.

## Figures and Tables

**Table 1:** The biomass of seeds, shoots, and roots of the WT and *P* plants under the Ri and Cd treatments.

Varieties	Indicator	CK	Ri	Cd	Ri+Cd
WT	Height (cm)	102.0 ± 2.5 ^a^	99.2 ± 1.4 ^a^	104.0 ± 1.4 ^a^	100.8 ± 1.7 ^a^
	Root length (cm)	32.8 ± 2.8 ^a^	29.6 ± 2.7 ^a^	36.8 ± 2.7 ^a^	36.2 ± 3.3 ^a^
	Seeds (g)	19.4 ± 1.4 ^c^	21.6 ± 0.6 ^bc^	31.6 ± 0.5 ^a^	28.4 ± 3.3 ^ab^
	Shoots (g)	29.7 ± 3.1 ^b^	29.2 ± 1.5 ^b^	42.6 ± 1.6 ^a^	30.9 ± 3.9 ^b^
	Roots (g)	3.8 ± 0.7 ^a^	3.8 ± 1.0 ^a^	4.4 ± 0.7 ^a^	4.1 ± 0.6 ^a^
*P*	Height (cm)	94.4 ± 0.9 ^a^	93.3 ± 1.6 ^a^	99.2 ± 1.9 ^a^	92.8 ± 1.3 ^a^
	Root length (cm)	35.6 ± 1.5 ^b^	44.0 ± 2.1 ^a^	37.8 ± 1.3 ^b^	38.0 ± 2.0 ^b^
	Seeds (g)	22.1 ± 1.5 ^b^	21.8 ± 1.5 ^b^	29.3 ± 1.6 ^a^	21.1 ± 2.1 ^b^
	Shoots (g)	36.1 ± 4.2 ^a^	33.8 ± 3.2 ^a^	35.2 ± 2.4 ^a^	28.3 ± 3.0 ^a^
	Roots (g)	3.4 ± 0.4 ^ab^	2.6 ± 0.1 ^b^	3.7 ± 0.5 ^ab^	4.5 ± 0.8 ^a^

“WT” indicates wild-type rice. “*P*” indicates *osnramp5* mutant. “CK” indicates wild-type rice or *osnramp5* mutant grown under normal conditions; “Ri” indicates wild-type rice or *osnramp5* mutant inoculated with Ri; “Cd” indicates wild-type rice or *osnramp5* mutant subjected to Cd stress; “Ri+Cd” indicates wild-type rice or *osnramp5* mutant inoculated with Ri under Cd exposure. For the data (means ± SEs, *n* = 5), different letters (a, b, c) indicate significant differences (*p* < 0.05, Duncan’s test).

**Table 2:** The uptake of K, Ca, Fe, and Zn of the WT and *P* rice plants under the Ri and Cd treatments.

Varieties	Elements	Different Parts	CK	Ri	Cd	Ri+Cd
WT	K g/kg	Grain	3.6 ± 0.2 ^b^	3.8 ± 0.1 ^b^	4.3 ± 0.3 ^a^	4.6 ± 0.4 ^a^
		Shoot	25.2 ± 3.9 ^a^	29.4 ± 7.3 ^a^	27.2 ± 3.1 ^a^	30.0 ± 0.8 ^a^
		Root	1.6 ± 0.0 ^c^	2.1 ± 0.1 ^bc^	2.5 ± 0.7 ^ab^	2.9 ± 0.5 ^a^
	Ca g/kg	Grain	1.7 ± 0.3 ^b^	2.0 ± 0.3 ^ab^	1.9 ± 0.2 ^ab^	2.1 ± 0.1 ^a^
		Shoot	7.7 ± 0.8 ^c^	13.1 ± 1.7 ^a^	10.1 ± 0.7 ^b^	9.2 ± 1.2 ^bc^
		Root	14.0 ± 0.7 ^a^	13.4 ± 0.7 ^a^	11.6 ± 2.0 ^a^	12.4 ± 1.6 ^a^
	Fe g/kg	Grain	0.0 ± 0.0 ^ab^	0.0 ± 0.0 ^b^	0.1 ± 0.0 ^a^	0.1 ± 0.0 ^a^
		Shoot	0.5 ± 0.2 ^b^	1.3 ± 0.5 ^a^	0.6 ± 0.1 ^b^	0.8 ± 0.1 ^b^
		Root	27.1 ± 5.6 ^a^	28.5 ± 4.0 ^a^	28.0 ± 1.5 ^a^	31.0 ± 4.5 ^a^
	Zn mg/kg	Grain	24.8 ± 4.4 ^a^	29.7 ± 5.2 ^a^	26.5 ± 1.7 ^a^	26.5 ± 0.3 ^a^
		Shoot	44.3 ± 3.6 ^b^	38.6 ± 11.5 ^b^	43.4 ± 14.4 ^b^	71.3 ± 1.7 ^a^
		Root	76.9 ± 12.0 ^b^	78.0 ± 15.3 ^b^	81.5 ± 24.7 ^b^	130.4 ± 33.2 ^a^
*P*	K g/kg	Grain	3.8 ± 0.4 ^c^	4.4 ± 0.2 ^b^	4.4 ± 0.2 ^b^	4.8 ± 0.1 ^a^
		Shoot	28.0 ± 1.5 ^b^	30.4 ± 4.5 ^ab^	29.9 ± 1.3 ^ab^	33.9 ± 1.8 ^a^
		Root	3.0 ± 0.3 ^b^	2.4 ± 0.5 ^c^	2.6 ± 0.2 ^bc^	4.1 ± 0.2 ^a^
	Ca g/kg	Grain	2.0 ± 0.1 ^a^	1.8 ± 0.2 ^a^	2.1 ± 0.2 ^a^	1.9 ± 0.3 ^a^
		Shoot	8.1 ± 1.6 ^b^	9.9 ± 0.8 ^a^	8.5 ± 1.1 ^ab^	8.6 ± 0.1 ^ab^
		Root	10.4 ± 1.3 ^b^	10.5 ± 1.2 ^b^	13.6 ± 2.4 ^a^	12.0 ± 2.0 ^ab^
	Fe g/kg	Grain	0.1 ± 0.0 ^a^	0.1 ± 0.0 ^a^	0.1 ± 0.0 ^a^	0.1 ± 0.0 ^a^
		Shoot	0.7 ± 0.1 ^a^	0.5 ± 0.0 ^a^	0.6 ± 0.3 ^a^	0.7 ± 0.1 ^a^
		Root	24.1 ± 6.8 ^b^	19.6 ± 2.6 ^b^	32.5 ± 3.5 ^a^	24.4 ± 6.6 ^ab^
	Zn mg/kg	Grain	33.0 ± 2.8 ^a^	35.1 ± 4.1 ^a^	35.0 ± 7.6 ^a^	35.7 ± 5.3 ^a^
		Shoot	34.9 ± 3.6 ^ab^	45.5 ± 12.4 ^a^	29.5 ± 8.2 ^b^	30.2 ± 4.6 ^b^
		Root	68.2 ± 16.9 ^b^	116.8 ± 37.3 ^a^	82.6 ± 47.0 ^ab^	99.3 ± 22.2 ^ab^

For the data (means ± SEs, *n* = 5), different letters (a, b, c) indicate significant differences (*p* < 0.05, Duncan’s test). The abbreviations used in Table 2 were same as that of Table 1.

**Table 3:** The primer sequences of qRT-PCR.

Primer Name	Forward (5′–3′)	Reverse (5′–3′)
*OsACTIN*	CAACACCCCTGCTATGTACG	CATCACCAGAGTCCAACACAA
*OsNRAMP1*	CATCGGCATCGTGCTGTTC	TGGCTACCTGTGCTTTCTCG
*OsNRAMP5*	AGCAGCAGTAAGAGCAAGATGG	GGGGAGGTCGTTGTGGATG
*OsIRT1*	GAACCGCGTCGTCGTTCAG	CCATCCCCTCGAACATCTGG
*OsZIP3*	AAGGTGTAGGTTTGGGTGGTTG	TCCTGCTGAGGCTGAGTTGAAG
*OsZIP7*	CCTTGCAATCTGGGCCTGAA	CAGATTAGTCTCACGCCCATGA
*OsCAX1a*	TGTGGGTGTTCGCTCTTAGT	GTGCAATCTGCTCTGTGAGG

## Data Availability

Data are contained within the article and Supplementary Materials.

## Supplementary Materials

The following supporting information can be downloaded at https://www.mdpi.com/article/10.3390/ijms26041464/s1.

## Abbreviations

The following abbreviations are used in this manuscript:

AMF	arbuscular mycorrhizal fungi
Cd	cadmium
CAX	cation^2+^/H^+^ exchanger
HMA	heavy metal ATPase
IRT	iron-regulated transporter
LCT	low-affinity cation transporter
NRAMP	natural resistance-associated macrophage protein
*P*	*osnramp5* mutant
*P*+Ri	*P* with Ri inoculation
*P*+Cd	*P* with Cd treatment
*P*+Ri+Cd	*P* with Ri inoculation and Cd treatment
Ri	*Rhizophagus intraradices*
WT	Wild-type rice
WT+Ri	WT with Ri inoculation
WT+Cd	WT with Cd treatment
WT+Ri+Cd	WT with Ri inoculation and Cd treatment
ZIP	Zn-regulated transporter
