# Supplementary material for: The Effect of Rhizophagus intraradices on Cadmium Uptake and OsNRAMP5 Gene Expression in Rice
Source: Int J Mol Sci. 2025 Feb 10;26(4):1464. doi: 10.3390/ijms26041464 (PMC11855883; doi:10.3390/ijms26041464)
Supplement: Supplementary file 1 [file ijms-26-01464-s001.zip › ijms-3460868-supplementary.pdf]

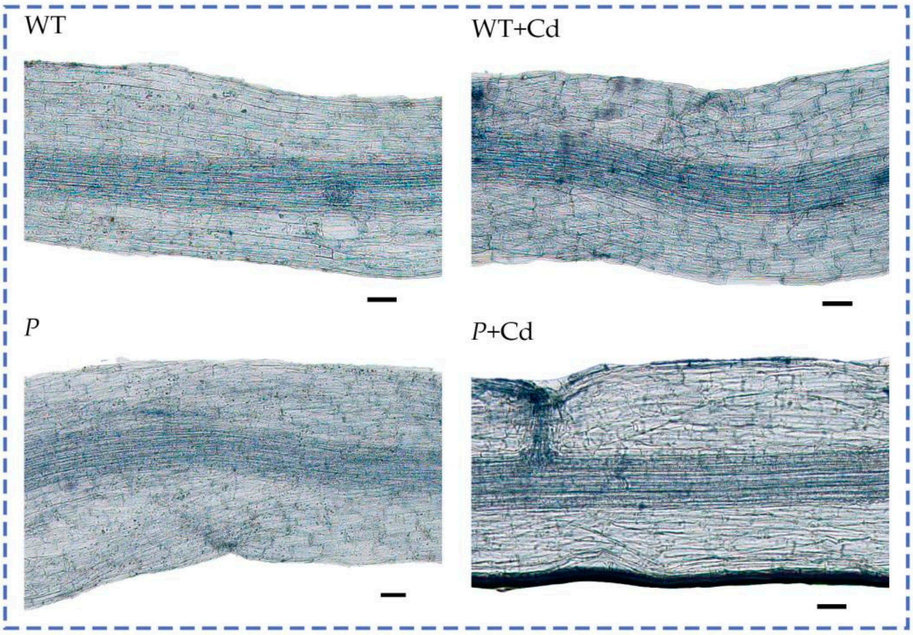

Figure S1. The colonization picture of WT and *P* plant roots under different treatments (Bar=50 μm).

"WT" indicates wild type rice grew under normal conditions; "*P*" indicates *osnramp5* mutant grew under normal conditions; "WT+Cd" indicates wild type rice was subjected to Cd stress, "*P*+Cd" *osnramp5* mutant was subjected to Cd stress.

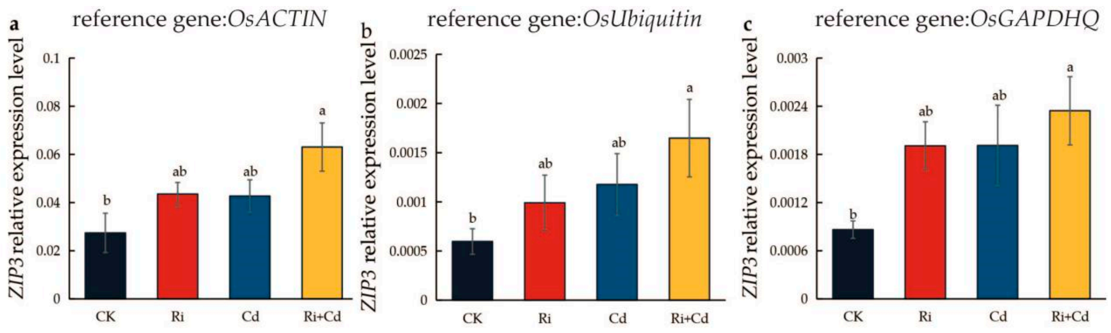

Figure S2. Expression levels of the *OsZIP3* in WT inoculated with or without AMF under Cd stress using three reference genes

Leaves were harvested separately for qRT-PCR analysis. Fold changes of the gene expression were normalized against the reference gene *OsACTIN* (a), *OsUbiquitin* (b) and *OsGAPDH* (c) calculated by the formula ($2^{(-\Delta Ct)}$). "CK" indicates wild type rice grew under normal conditions; "Ri" indicates wild type rice was inoculated with Ri; "Cd" indicates wild type rice was subjected to Cd stress, "Ri+Cd" indicates wild type rice was inoculated with Ri under Cd exposure. Data (means ±SE, n = 5), different letters (a, b, c, d, etc) indicate significant differences ($p < 0.05$, Duncan's test)

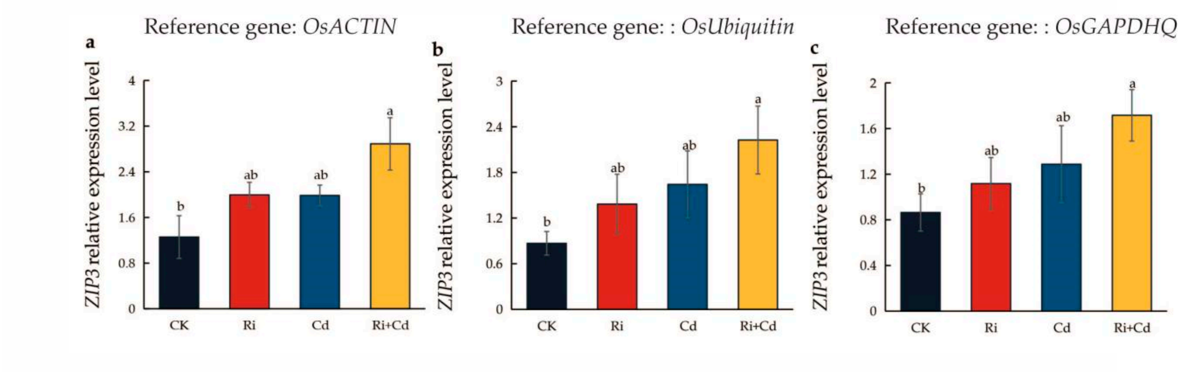

Figure S3. Expression levels of the *OsZIP3* in WT inoculated with or without AMF under Cd stress calculated by the formula $2^{(-\Delta\Delta Ct)}$

Leaves were harvested separately for qRT-PCR analysis. Fold changes of the gene expression were normalized against the reference gene *OsACTIN* (a), *OsUbiquitin* (b) and *OsGAPDH* (c) calculated by the formula ($2^{(-\Delta\Delta Ct)}$). Relative gene expression was calculated by the formula $2^{(-\Delta\Delta Ct)}$. the first ΔCT is the difference in threshold cycle between the target and reference genes: $\Delta CT = Ct_{\text{target gene}} - Ct_{\text{internal reference gene}}$); $\Delta\Delta CT = \Delta Ct_{\text{target gene}} - \Delta Ct_{\text{internal reference gene}}$, $2^{(-\Delta\Delta Ct)}$ = (Power (2, − ΔΔCT), Ct: computerized tomography. “CK” indicates wild type rice grew under normal conditions; “Ri” indicates wild type rice was inoculated with Ri; “Cd” indicates wild type rice was subjected to Cd stress, “Ri+Cd” indicates wild type rice was inoculated with Ri under Cd exposure. Data (means ±SE, n= 5), different letters (a, b, c, d, etc) indicate significant differences ($p < 0.05$, Duncan's test).

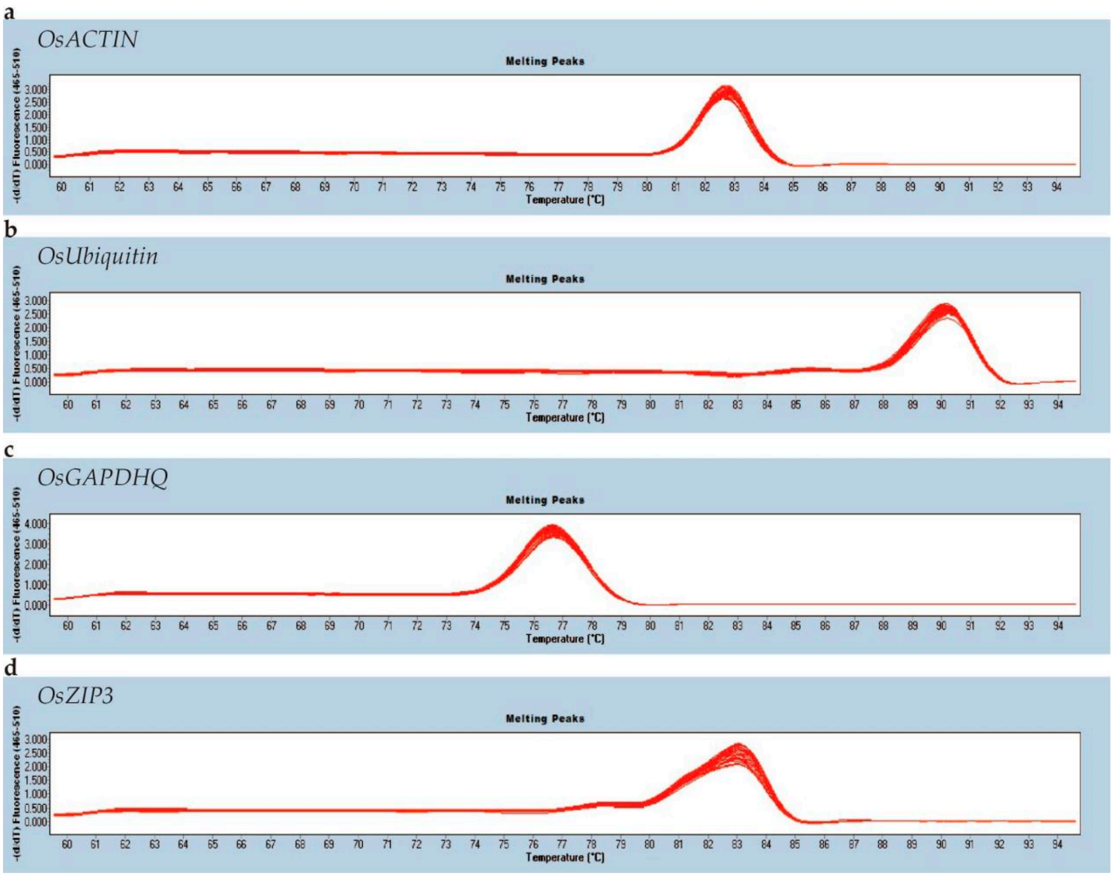

Figure S4. Analysis of melting curves for *OsACTIN* (a), *OsUbiquitin* (b), *OsGAPDH* (c) and *OsZIP3* (d)

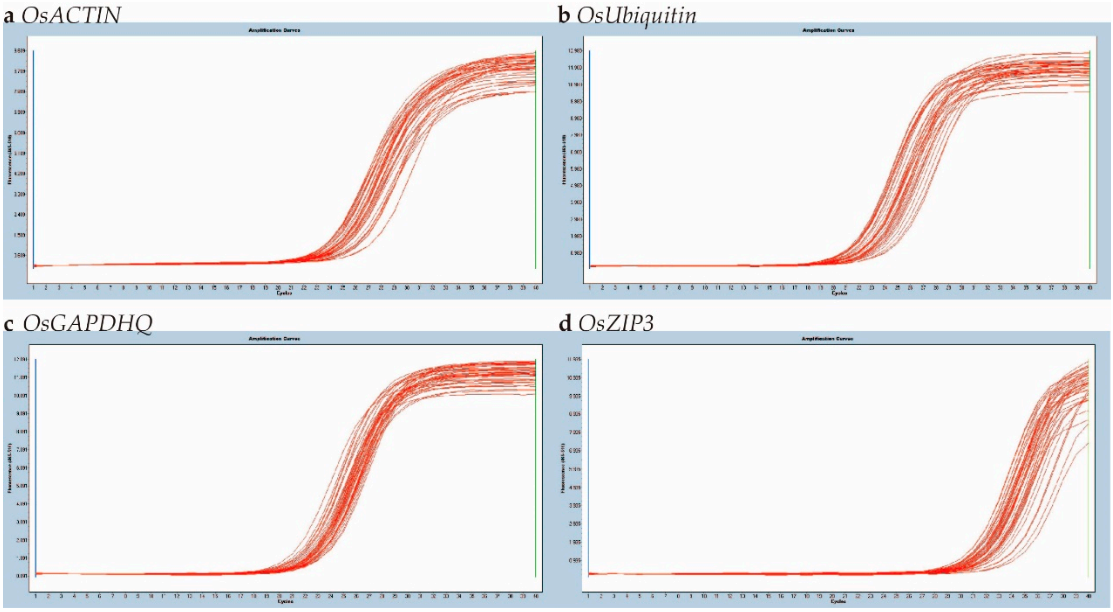

Figure S5. The amplified curve of *OsACTIN* (a), *OsUbiquitin* (b), *OsGAPDH* (c) and *OsZIP3* (d)

Table S1. the primer sequences of qRT-PCR.

Primer name	Forward (5'-3')	Reverse (5'-3')
OsUbiquitin	ATCACGCTGGAGGTGGAGT	AGGCCTTCTGGTTGTAGACG
OsGAPDH	CTGATGATATGGACCTGAGTCTACTTTT	CAACTGCACTGGACGGCTTA
